# Mesoporous Palladium N,N’-Bis(3-Allylsalicylidene)o-Phenylenediamine-Methyl Acrylate Resins as Heterogeneous Catalysts for the Heck Coupling Reaction

**DOI:** 10.3390/ma12162612

**Published:** 2019-08-16

**Authors:** Claudio Mella, Cecilia C. Torres, Gina Pecchi, Cristian H. Campos

**Affiliations:** 1 Depto. Físico-Química, Facultad de Ciencias Químicas, Universidad de Concepción, Edmundo Larenas 129, Concepción 4070371, Chile; 2 Depto. de Ciencias Químicas, Facultad de Ciencias Exactas, Universidad Andrés Bello, Sede Concepción, Autopista Concepción-Talcahuano 7100, Talcahuano 4300866, Chile; 3 Millenium Nuclei on Catalytic Processes towards Sustainable Chemistry (CSC), Santiago 8340518, Chile

**Keywords:** porous polymer, metal chelate monomer, palladium coordinated polymer

## Abstract

Palladium N,N’-bis(3-allylsalicylidene)o-phenylenediamine complex (PdAS) immobilized onto mesoporous polymeric methyl acrylate (MA) based resins (PdAS(x)-MA, x = 1, 2, 5, or 10 wt.%) were successfully prepared as heterogeneous catalysts for the Heck reaction. The catalysts were synthesized via radical suspension polymerization using PdAS as a metal chelate monomer, divinylbenzene and MA as co-monomers. The effect of the PdAS(x) content on the physicochemical properties of the resins is also reported. The catalysts were characterized by using a range of analytical techniques. The large surface area (>580 m^2^·g^−1^) and thermal stability (up to 250 °C) of the PdAS(x)-MA materials allows their application as catalysts in the C–C coupling reaction between iodobenzene and MA in the presence of trimethylamine at 120 °C using DMF as the solvent. The PdAS(10)-MA catalyst exhibited the highest catalytic performance with no significant catalytic loss being observed after five reuses, thereby indicating excellent catalyst stability in the reaction medium.

## 1. Introduction

The use of palladium coordination complexes as homogeneous catalysts for a large number of low scale and industrial process is widely known, and in particular for the preparation of various important organic building blocks [1,2,3]. Indeed, the formation of new carbon–carbon bonds through the catalyzed carbon-coupling reactions of a large variety of starting materials are key to the area of organic chemistry [4,5,6]. Additionally, the requirement of tolerance to almost any unprotected sensitive functional groups [7,8] increase their recognized importance as some of these C–C coupling reactions, such as the Heck, Suzuki and Negishi reactions, have been recognized with the 2010 Nobel Prize [4,9]. Even though homogeneous palladium complexes have been reported the most important catalytic systems [5,6], the major drawback is related with their poor recovered capacity and the difficulty of separating it from the reaction medium, with several environmental consequences. This latter problem, together with the new vision of green chemistry procedures, is the reason of the current efforts to heterogenize homogeneous catalysts [10,11]. To achieve this challenge, one procedure has been the immobilization of the coordination compounds on insoluble matrixes to obtain heterogeneous catalysts, improving their mechanically stability to be reused [12,13,14,15,16].

Due to the enhanced stability, the covalent immobilization pathway has therefore proven to be a particularly efficient option for the preparation of heterogeneous metal complex-based catalysts [17]. To date, different insoluble materials have been reported for this purpose, including amorphous or mesostructured silica materials [18], inorganic-organic hybrid materials [19], and polymeric supports [20,21]. With regard to the polymeric support, the most commonly employed material is the lightly cross-linked Merrifield resin [20,22,23], which exhibits a good thermal stability and a surface that acts as a simple anchoring point for metal complex immobilization; however, such systems tend to exhibit a low surface area, which limits their use in heterogeneous systems [24]. To address this issue, a range of methodologies for the preparation of polymeric materials with high surface areas have been reported based on the use of metal chelate monomers (MCM) as co-monomers [25,26]. For example, porous organic polymers (POPs) [27], polymers synthesized by high internal phase emulsion (Poly-HIPEs) [28,29], and cross-coupling polymers [30,31] have been reported. The main disadvantages in using such methodologies to produce immobilized metal-complexes on heterogeneous matrices is the expensive and complex synthetic routes required [21]. Therefore, cross-linked polymers with a high divinylbenzene (DVB) content of DVB open the possibility of providing large surface area materials when it is synthesized by suspension polymerization in presence of a porogen agent allow to obtain materials with tunable properties. Since suspension polymerization allows tuning of the morphology, surface area, and pore size through economical and simple synthetic procedures [21,32,33], it is commonly employed in the production of polymer beads with applications in chromatographic separation media (e.g., ion exchange resins) and as supports for enzyme or metal-complex immobilization [34,35].

The current necessity for new coordination compounds bearing non-expensive and stable ligands as catalysts for carbon coupling reactions has led to the design and synthesis of various nitrogen-based compounds bearing amine [14], oxime [36,37], quinolone [14], pyridine [38], and imine [14,39,40] functional groups. In particular, imines synthesized by the condensation of salicyl-aldehyde with an aliphatic or an aromatic diamine are commonly known as SALEN or SALOPHEN ligands, respectively, and these ligands can be employed as N_2_O_2_ ligands for the preparation of palladium-based catalysts. Recently, homogeneous/immobilized Pd-SALEN and -SALOPHEN catalysts have been reported as excellent catalysts for use in the Mizoroki–Heck [31,41,42,43,44] and Suzuki–Miyaura [44,45,46,47,48] C–C coupling reactions, due to their high selectivity, air- and moisture-stability, and relatively low toxicity. 

Thus, we herein report the synthesis of highly crosslinked mesoporous resins for immobilization of the N,N’-bis(3-allyl-salicylidene)o-phenylenediamine palladium(II) (PdAS) complex to provide heterogeneous catalysts for application in the Heck coupling reaction. The novelty of this work is the design of a synthetic strategy that includes the incorporation of PdAS as a MCM in the material formulation. In addition, the one-step radical suspension polymerization of PdAS using divinylbenzene (DVB) and methyl acrylate (MA) as co-monomers, in addition to 2,2′-azobis(2-methylpropionitrile) (AIBN) as a radical initiator, is also reported. The effect of the PdAS loading on the physicochemical and catalytic properties of the resulting PdAS(x)-MA (x = 1, 2, 5, or 10 wt.%) resins as catalysts for the Heck reaction of iodobenzene (I-Ph) and MA to produce methyl cinnamate (MCIN) is also assessed. For comparison, palladium(II) acetate (Pd(CH_3_CO_2_)_2_) and N,Nʹ-bis(salicylidene)o-phenylenediamine palladium(II) (Pd-SALOPHEN) complexes are also tested as homogeneous catalysts for the production of MCIN. Finally, we evaluate the operational stabilities of the most active catalyst over five consecutive catalytic cycles.

## 2. Materials and Methods

### 2.1. Chemicals and Reagents

Iodobenzene (I-Ph), sodium chloride (NaCl), palladium (II) acetate (Pd(CH_3_CO_2_)_2_), methanol (MeOH), absolute ethanol (EtOH), N,N-Dimethylformamide (DMF), diethyl ether (Et_2_O), dichloromethane (DCM), dodecane, and acetone were purchase from Merck^®^ (Kenilworth, NJ, USA) and used as received. Technical divinylbenzene (DVB, 90%), hydroxiethylcellulose (HEC), 3-allylsalicylaldehyde, o-phenylendiamine, methyl acrylate (MA), triethylamine (TEA) and, 2,2’-azobis(isobutyronitrile) (AIBN) were purchase from Sigma-Aldrich^®^ (Saint Louis, MO, USA). TEA was distilled at reduced pressure with CaH_2_ and MA at normal pressure in presence of p-tert-butylcatechol as polymerization inhibitor. AIBN was purified by recrystallization from methanol.

### 2.2. Synthesis of the Schiff Base Ligands and Pd(II) Complexes

The N,N’-bis(3-allylsalicylidene)o-phenylenediamine (AS) and N,N’-bis(salicylidene)o-phenylenediamine (SALOPHEN) ligands were synthesized according to previously described literature methods [46]. The desired PdAS and Pd-SALOPHEN complexes were then prepared from Pd(CH_3_CO_2_)_2_ (a Pd(II) precursor) and the appropriate AS or SALOPHEN ligand, as described in the literature [49,50]. Characterization details for the ligands and the Pd(II)-complexes can be found in the supplementary material.

### 2.3. Synthesis of the PdAS(x)-MA, (x = 1, 2, 5 or 10 wt.%) Resins

The Pd-MAS(x)-MA resins were prepared in a three-neck reactor using different quantities of PdAS with careful control of the temperature, stirring speed, and N_2_ flow. Table 1 shows the nominal amounts of DVB, MA, PdAS, and AIBN employed. The co-monomers (DVB and MA) and the initiator (AIBN) were mixed and stirred, after which toluene was added (monomers:toluene, 1:3 volume ratio). The mixture was suspended over an aqueous phase containing 20 wt.% NaCl and 0.2 wt.% HMC, subjected to a flow of N_2_ (10 mL min^−1^), and heated at 70 °C for 14 h then at 88 °C for 4 h with mechanical stirring (400 rpm). The obtained brown solids were washed with water for 48 h and subsequently with dichloromethane (DCM) for 24 h using a Soxhlet apparatus. Finally, the obtained coordinated polymers denoted PdAS(x)-MA, where x = 1, 2, 5, or 10 wt.% PdAS, were dried at 83 °C in a vacuum oven.

### 2.4. Characterization

Thermogravimetry analysis (TG/DTG) experiments were performed at a heating rate of 10 °C min^−1^ up to 1000 °C with a N_2_ flow of 8.0 mL·min^−1^ (209F1 Iris, NETZSCH TG, Selb, Germany), using a sample mass of 3.0 mg. The nitrogen adsorption isotherms were measured using a Micromeritics equipment model TriStar II Series 2 instrument (Norcross, GA, USA) at −196 °C on samples previously treated by degasification for 3 h at 120 °C. The specific surface areas and pore size distributions were calculated using the Brunauer–Emmett–Teller (BET) and Barrett–Joyner–Halenda (BJH) methods, respectively. The catalyst morphologies were determined by scanning electron microscopy (SEM, Vega 3 LMU, TESCAN, Brno, Czech Republic) coupled with energy-dispersive X-ray spectroscopy (EDS). NMR spectroscopy (^13^C decoupled, ^1^H, C–H correlation, and solid-state cross polarized magic angle spinning (CP/MAS)) was carried out on a Bruker ASCEND 400 MHz spectrometer (Billerica, MA, USA) with an inverse multinuclear probe. The diffuse reflectance spectroscopy (DRS) UV–vis measurements were carried out on a Thermo Scientific Evolution 260Bio UV–vis spectrophotometer (Waltham, MA, USA). Liquid phase UV–vis spectra were recorded on a Spectroquant Pharo 300 instrument (Thomas Scientific, Swedesboro, NJ, USA), using DCM as the solvent. Palladium quantification was carried out by inductively coupled plasma optical emission spectrometry (ICP-OES) using a Spectro Arcos instrument (Kleve, Germany). Samples were previously digested in a mixture of concentrated HNO_3_ and H_2_SO_4_ under microwave irradiation using a Milestone Srl Ethos Easy (Brøndby Kommune, Denmark) apparatus prior to dilution for analysis. Pd quantification of the post-reaction liquid phase was measured by Atomic Absorption Spectroscopy (AAS) using a Thermo Scientific iCE 3000 Series AA-spectrometer (Waltham, MA, USA). XPS measurements were performed using a Surface Analysis Station 1 electron spectrometer (XPS RQ300/2, Staib Instrumente GmbH, Langenbach, Germany) equipped with a hemispherical electron analyzer DESA 150 detector/2700V and non-monochromatic Al Kα (1486.6 eV) X-ray source.

### 2.5. Catalytic Activity

The catalytic performance of each Pd-based catalyst was evaluated in the carbon-coupling reaction of I-Ph and MA to produce MCIN. Molar ratios of I-Ph:Pd = 2000:1 and I-Ph:MA:TEA = 1:1.25:2 were employed. The reaction was carried out in a Parr type semi-batch reactor with a reactant pre-chamber (Scheme S1) at 120 °C under an Ar pressure of 5.5 bar using DMF as solvent. The total reaction mixture volume was 50 mL, and magnetic stirring was sustained throughout at a rate of 770 rpm. The PdAS(x)-MA catalyst was mixed with I-Ph, DMF, and dodecane then heated at 120 °C under an inert atmosphere, after which time MA and TEA were added from the pre-chamber. The conversion of I-Ph was followed by GC (Perkin Elmer Clarus 680 GC equipped with an Elite-MS5 capillary column, Waltham, MA, USA) using dodecane as an internal standard (908 µL dodecane, 0.8 mmol). Sample aliquots were collected at the desired reaction times, mixed with water (1 mL), extracted with Et_2_O (1 mL), and washed with brine (1 mL). The organic layer was then analyzed by GC. For all catalytic tests the first aliquot was taken at the moment of MA and TEA addition. The retention time of the product was confirmed with a commercial standard product. The total conversion and selectivity were calculated as

(1) $$\%Conversion=\;\frac{{\left[I-Ph\right]}_{i}-{\left[I-Ph\right]}_{t}}{{\left[I-Ph\right]}_{i}}\times 100$$

(2) $$\%Selectivity\;\left(MCIN\right)=\;\frac{{\left[MCIN\right]}_{t}}{{\left[I-Ph\right]}_{i}-{\left[I-Ph\right]}_{t}}\times 100$$

Hence, turnover frequency (TOF) was calculated at 5% of conversion level as

(3) $$TOF\left(h^{-1}\right)=\;\frac{mol\;I-Ph\;consumed}{mol\;Pd\cdot time}$$

Reusability and leaching tests were also performed for the most active, selective, and operationally stable catalyst. For the reusability test, following the initial catalytic run, the catalyst was collected by direct filtration from the reactor, and the recovered catalyst was subjected to the subsequent catalytic cycle under identical conditions. The leaching test was carried out using the Sheldon test with fresh and reused (after 5 cycles) catalysts [44,46,47]. For the fresh catalyst, the reaction mixture was maintained for 5 min, the catalyst was separated by hot filtration, the homogeneous solution was recharged with volatile reactants (MA and TEA), and the reaction was carried out under identical reaction conditions. For the reused catalyst (after 5 cycles), the same methodology was employed after 240 min of reaction. In both experiments, the quantity of palladium leached from the catalysts to the reaction medium was analyzed by AAS after catalyst separation.

## 3. Results and Discussion

### 3.1. Thermogravimetric Analysis (TGA)

**Figure 1:**
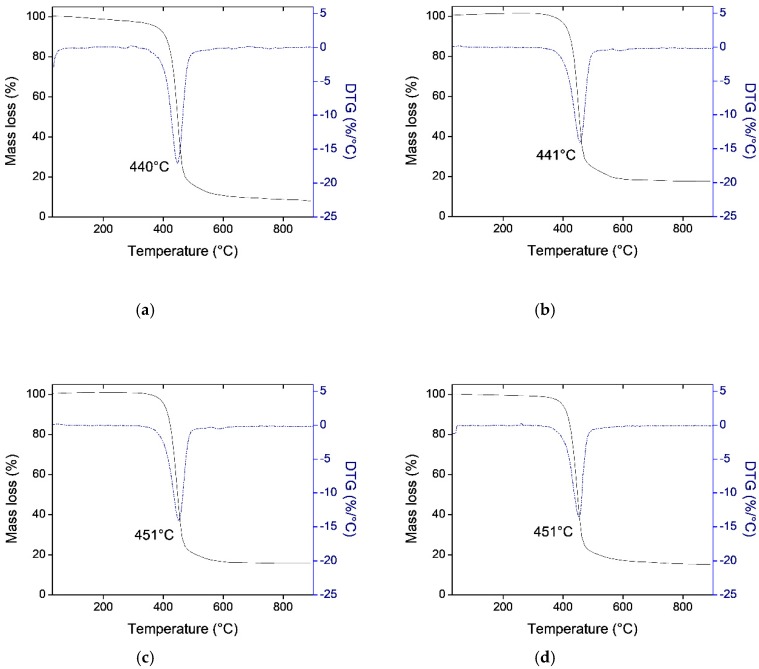
Thermogravimetric profiles of PdAS(x)-MA catalysts. (**a**) PdAS(1)-MA, (**b**) PdAS(2)-MA, (**c**) PdAS(5)-MA, and (**d**) PdAS(10)-MA.

The thermal stabilities of the PdAS(x)-MA catalysts are outlined in Figure 1, where it can be seen that the synthesized polymers exhibited the desorption of physisorbed species between 90 and 180 °C, which was attributed to solvent evaporation (weight loss ~1%). The most important weight loss of 79% appeared between 380 and 510 °C, and this was attributed to the thermal depolymerization/decomposition process [51]. The absence of any significant weight loss below 250 °C therefore confirms the high thermal stability of the PdAS(x)-MA polymers, and confirms their suitability for use as heterogeneous catalysts in the carbon-coupling Heck reaction due to the relatively high temperatures required for this transformation. 

### 3.2. Scanning Electron Microscopy (SEM) Coupled with Energy-Dispersive X-ray Spectroscopy (EDS)

**Figure 2:**
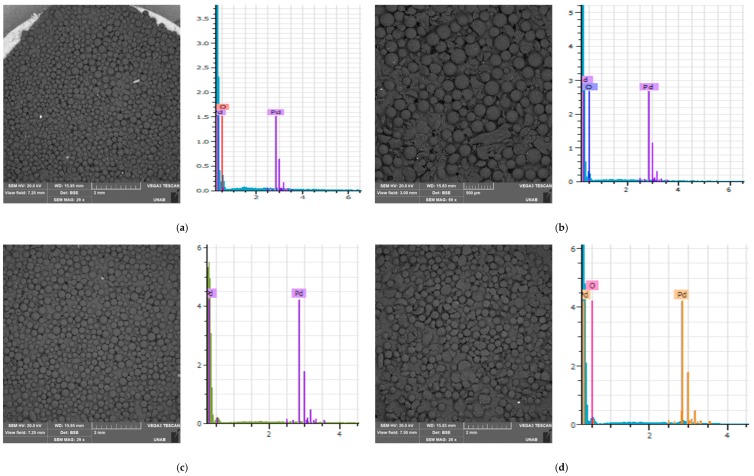
SEM energy-dispersive X-ray spectroscopy (SEM-EDS) micrographs analysis. (**a**) Pd-AS(1)-MA (**b**) Pd-AS(2)-MA (**c**) Pd-AS(5)-MA, and (**d**) Pd-AS(10)-MA.

SEM characterization of the PdAS(x)-MA catalysts showed well-defined polymeric microspheres (Figure 2), which indicates the importance of toluene in determining the hydrophobic properties of the materials during synthesis, with the formation of well-defined and uniform oil droplets being observed [52]. The diameter particle size distribution (Figure S1) obtained by counting more than 600 particles directly from the SEM micrographs gave a narrow size distribution, and allowed estimation of the standard deviation of the particle size, as shown in Table 1. As indicated, an increase in the mean diameter was observed from 184 to 272 µm, which was attributed to the increase of PdAS MCM in the resin formulation. The EDS results are shown as an inset in the SEM micrographs of Figure 2 and confirm the presence of Pd on the surfaces of the synthesized PdAS(x)-MA resins.

### 3.3. Nitrogen Adsorption-Desorption Isotherms at −196 °C

**Figure 3:**
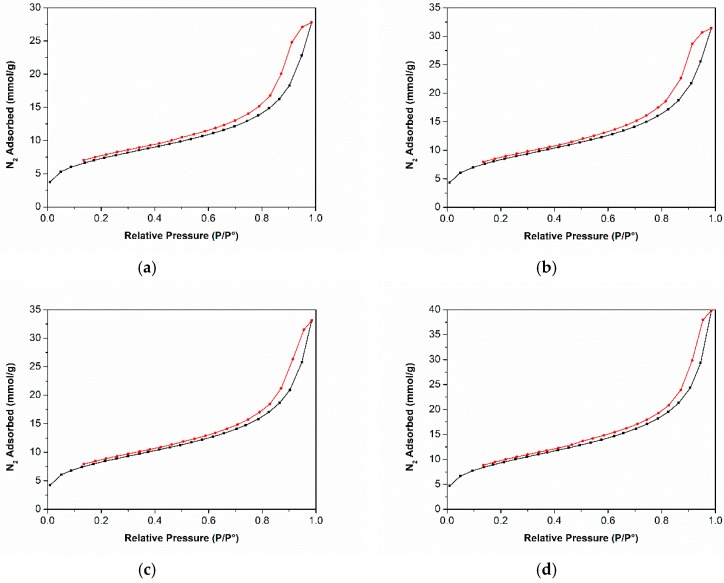
N_2_ adsorption-desorption isotherm at –196 °C for the PdAS(x)-MA catalysts. (**a**) Pd-AS(1)-MA, (**b**) Pd-AS(2)-MA, (**c**) Pd-AS(5)-MA, and (**d**) Pd-AS(10)-MA.

The N_2_ adsorption–desorption isotherms of the PdAS(x)-MA catalysts are shown in Figure 3, where type IV isotherms can be seen with a hysteresis loop at moderate to high pressures, thereby indicating the presence of mesoporous materials [53]. The specific Brunauer–Emmett–Teller (S_BET_) surface areas given in Table 1 indicate large values with a dependence regarding the PdAS content. More specifically, a low S_BET_ value was obtained for PdAS(1)-MA (586 m^2^·g^−1^), and this increased as the quantity of PdAS MCM was increased, giving a value of 754 m^2^·g^−1^ for the PdAS(10)-MA resin. This sustained increase in surface area is in line with a previous report by Lin et al. for High-DVB cross-linked polymeric beads where the S_BET_, the total pore area, and the total pore volume increased with increasing DVB content in styrene-DVB resins [54]. In this way, we suggest that PdAS could be polymerize as a cross-linking molecule mainly due to its two-polymerizable allyl groups.

### 3.4. CP/MAS ^13^C NMR Spectroscopy

**Figure 4:**
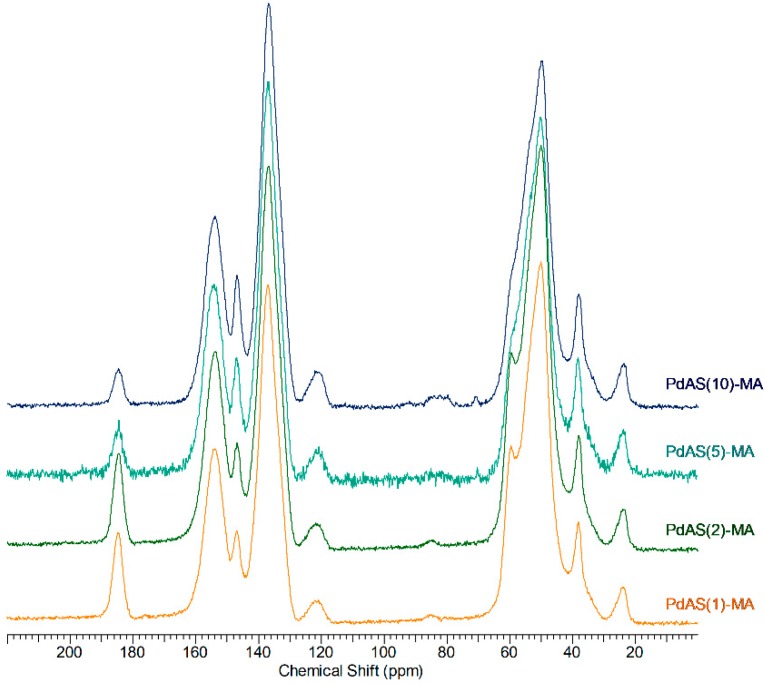
CP/MAS ^13^C NMR spectra of PdAS(x)-MA catalysts.

Figure 4 shows the CP/MAS ^13^C NMR spectra of the PdAS(x)-MA catalysts, where the main signals were found to correspond with the DVB component [55,56]. However, the absence of signals attributable to the PdAS coordination compound does not mean that it is not present, and indeed it is likely that these signals are masked by the aromatic DVB profile and the aliphatic backbone of the polymer. As shown, characteristic bands of the DVB ethyl benzene –CH_2_– and –CH_3_ moieties were observed at 33 and 18 ppm respectively, while a broad –CH_2_ signal originating from the backbone of the polymeric matrix was centered at 50 ppm [53], while those at 121–153 ppm were attributed to the aromatic carbon atoms of the DVB cross-linker. Only two signals originating from MA were detected, the first as a shoulder at 58 ppm (–OCH_3_), and the second at 184 ppm (C=O). In line with the DRS UV–vis analysis results (see below), the decrease of both signals can be associated with an increase in the PdAS content of PdAS(x)-MA. These results therefore confirm the successful incorporation of PdAS to replace MA in the synthesized PdAS(x)-MA polymers.

### 3.5. DRS UV–Vis Measurements

**Figure 5:**
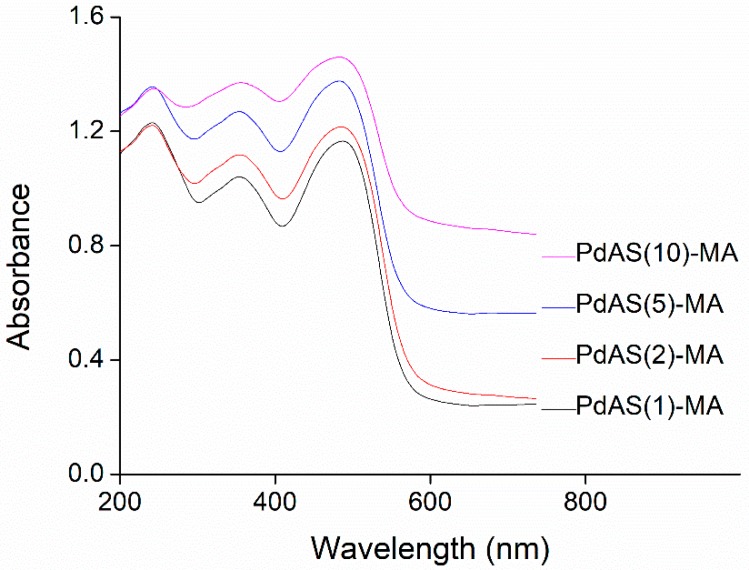
DRS UV–vis spectra for the PdAS(x)-MA catalysts.

Analysis by DRS UV–vis spectroscopy gave bands at 371 nm attributed to the n → π* transition, in addition to a second transition band at 498 nm attributed to the C-T/d-d transitions typical for a metal-coordinated center on a Schiff-Base metal complex (Figure 5) [57]. Moreover, the progressive increase in absorbance was attributed to an increase in Pd-complex immobilization, thereby confirming the successful incorporation of the PdAS MCM onto the resins structure.

### 3.6. ICP-OES

The content of Pd(II) in the prepared PdAS(x)-MA resins was then determined by atomic ICP-OES and the results are summarized in Table 2. Our analysis revealed that the real PdAS content in the resins decreased from ~100% incorporation for PdAS(1)-MA to ~50% in the case of PdAS(10)-MA. We note that during the suspension polymerization process, the various reagents (co-monomers, DVB, MA, PdAS MCM, and AIBN) were dissolved in toluene and dispersed in a water-NaCl-HEC mixture to produce drops of the co-monomer solution (dispersed phase) suspended in the aqueous dissolution (continuous phase), where radical polymerization results in the formation of the desired resins beads [35]. When polymerization takes place in these drops, an increased PdAS loading in the dispersed phase promotes the formation of highly cross-linked resins, as determined by characterization of the N_2_ isotherms. This result suggests that limitations in the diffusion of PdAS MCM during the suspension polymerization process could be responsible for the decreased palladium content in PdAS(x)-MA resins where x > 1.

### 3.7. X-ray Photoelectron Spectroscopy (XPS)

**Figure 6:**
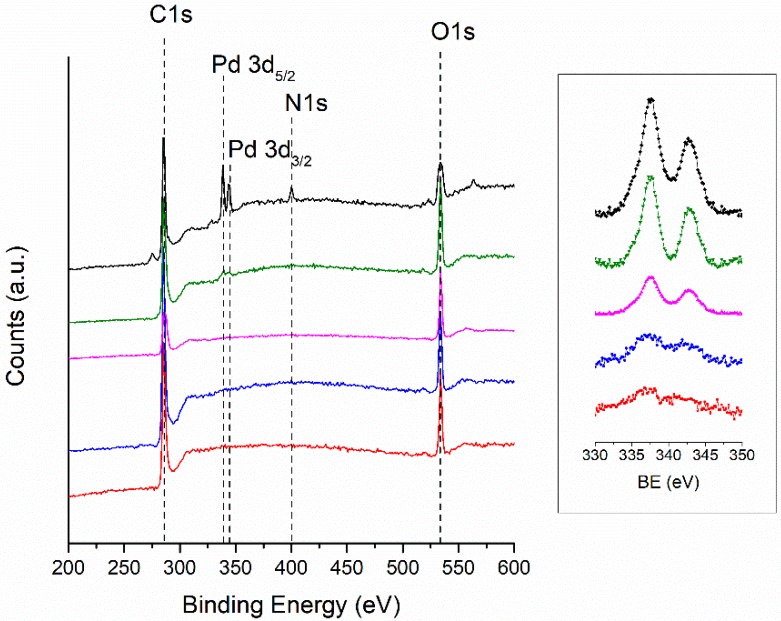
Survey XPS spectrum of the PdAS complex and PdAs(x)-MA catalysts (Pd 3d region; inset). Lines, PdAS complex (black), PdAS(1)-MA (red), PdAS(2)-MA (blue), PdAS(5)-MA (magenta) and PdAS(10)-MA (green).

XPS was subsequently carried out for the binding energies (BEs) of C 1s, N 1s, O 2p, and Pd 3d_5/2_ to determine the chemical environment of the surface palladium species in the PdAS(x)-MA polymers. For comparison, the XPS characterization of PdAS was also carried out. As shown in Figure 6, the wide range spectra of Pd 3d_5/2_ at 338.40 eV, C 1s at 285 eV, N 1s at 400.0 eV, and O 2p at 533.8 eV were obtained. In the case of PdAS, XPS analysis showed BEs for Pd 3d_5/2_ and 3d_3/2_ at 338.4 and 343.7 eV, respectively, while for N 1s the BE was detected at 400.0 eV. The Pd and N XPS contributions are therefore in line with the reported results for Pd-SALEN complexes [31,58]. As shown in the inset of Figure 6, the Pd contribution in the PdAS(x)-MA polymers can be more clearly observed upon magnification of the Pd core level region, where the BE of Pd 3d_5/2_ can be clearly seen at 338.40 eV with typical spin-orbital splitting (~5.30 eV) for Pd 3d_3/2_. The observed BEs of Pd 3d_3/2_ and Pd 3d_5/2_ therefore confirm the presence of palladium metal in the +2 oxidation state in all prepared catalysts [58]. This result confirms the successful immobilization of the PdAS MCM onto the resin structure.

### 3.8. Catalytic Activity

#### 3.8.1. Effect of PdAS Loading

The C–C coupling reaction of I-Ph with MA to produce MCIN was used to evaluate the catalytic activity of the mesoporous PdAS(x)-MA resins. Several experimental conditions have been reported for such a transformation, including a reaction temperature of 80–120 °C, different solvents (H_2_O, toluene, dimethylacetamide, DMF, and EtOH), and a range of bases (K_2_CO_3_, Na_2_CO_3_, Na(CH_3_CO_2_), pyrrolidine, and TEA) [59,60,61,62,63,64]. Thus, to evaluate the suitability of the PdAS(x)-MA resins as catalysts for the selected C–C coupling reaction, their catalytic activities were determined at 120 °C using TEA as the base and DMF as the solvent. A control experiment was also carried out in the absence of a catalyst over 10 h, and no I-Ph conversion or product formation was observed. In addition, the homogeneous Pd(CH_3_CO_2_)_2_ and Pd-SALOPHEN catalysts were evaluated under the same reaction conditions for comparison.

**Figure 7:**
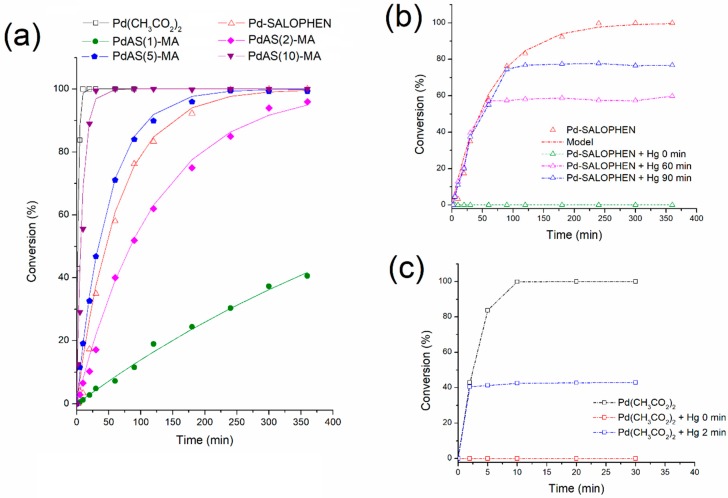
Kinetic profiles for the coupling of I-Ph with methyl acrylate to produce methyl cinnamate (MCIN) using the Pd-based catalysts. (**a**) Open square: Pd(CH_3_CO_2_)_2_; open triangle: Pd-SALOPHEN, fill circle: PdAS(1)-MA, fill diamond: PdAS(2)-MA, fill pentagon: PdAS(5)-MA, and fill inverse triangle: PdAS(10)-MA. (**b**) Hg(0) poisoning test for Pd-SALOPHEN catalyst and (**c**) Hg(0) poisoning test for Pd(CH_3_CO_2_)_2_ catalyst.

**Scheme 1:**
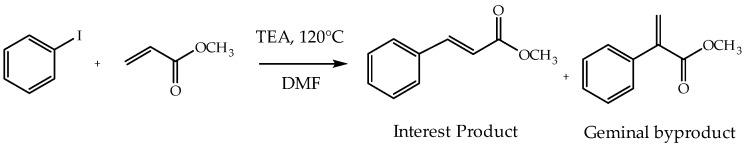
The Heck cross-coupling for I-Ph and MA to produce MCIN.

Figure 7a shows the I-Ph conversion-time profiles for the homogeneous Pd(CH_3_CO_2_)_2_ and Pd-SALOPHEN catalysts, and for the heterogeneous PdAS(x)-MA resins, where the catalytic activity decreased in the order of: Pd(CH_3_CO_2_)_2_ ~ PdAS(10)-MA >> PdAS(5)-MA ~ Pd-SALOPHEN > PdAS(2)-MA > PdAS(1)-MA. In addition, the obtained 100% selectivity toward the desired MCIN product indicated the absence of geminal coupling (see Scheme 1). This behavior has been attributed to the presence of TEA, which prevents the competitive I-Ph homo-coupling reaction taking place [65]. The time required to reach an iso-conversion of 5% I-Ph conversion as estimated from the exponential trend curves of the PdAS(x)-MA catalysts (Figure S3) indicates the same dependence on the immobilized PdAS content.

**Scheme 2:**
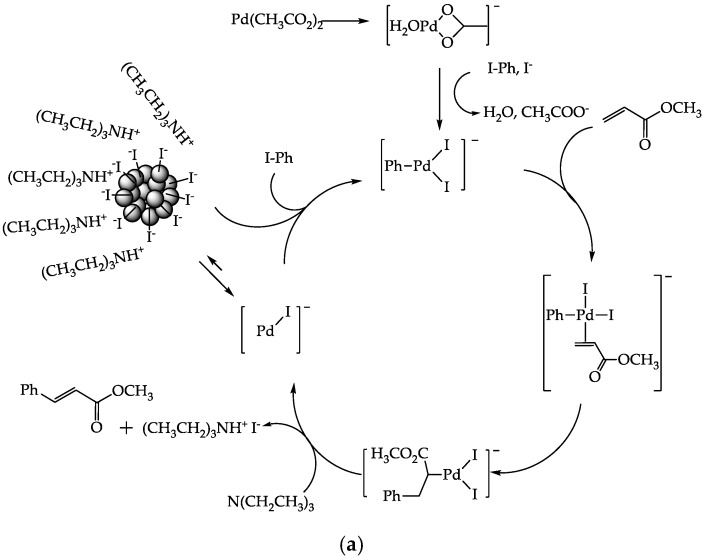

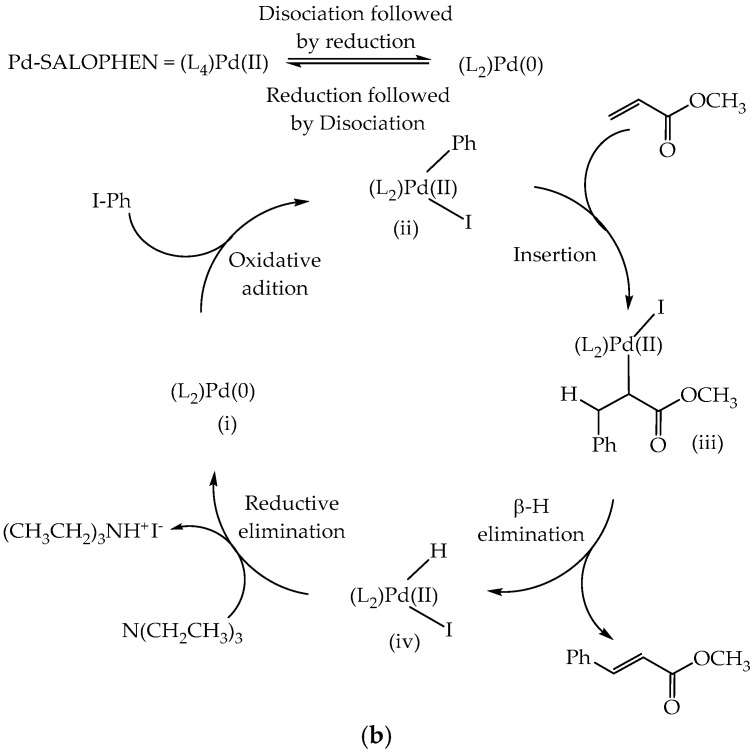
Proposed mechanism of the Heck reaction. (**a**) Mechanism for Pd(II)/Pd(0) adapted from [66] and (**b**) mechanism for tetradentate Pd(II) catalyst adapted from palladium(II)-porphyrin complex [71] as model homogeneous catalyst.

**Figure 8:**
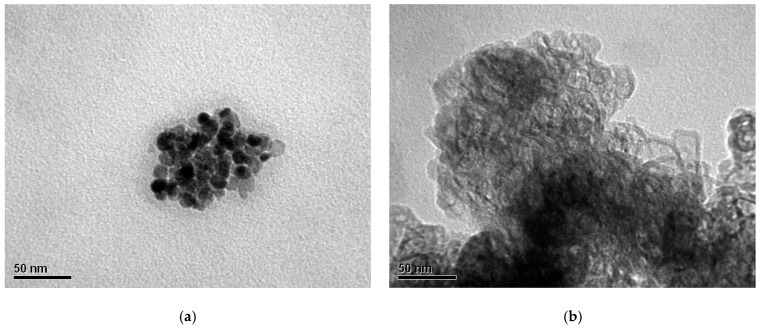

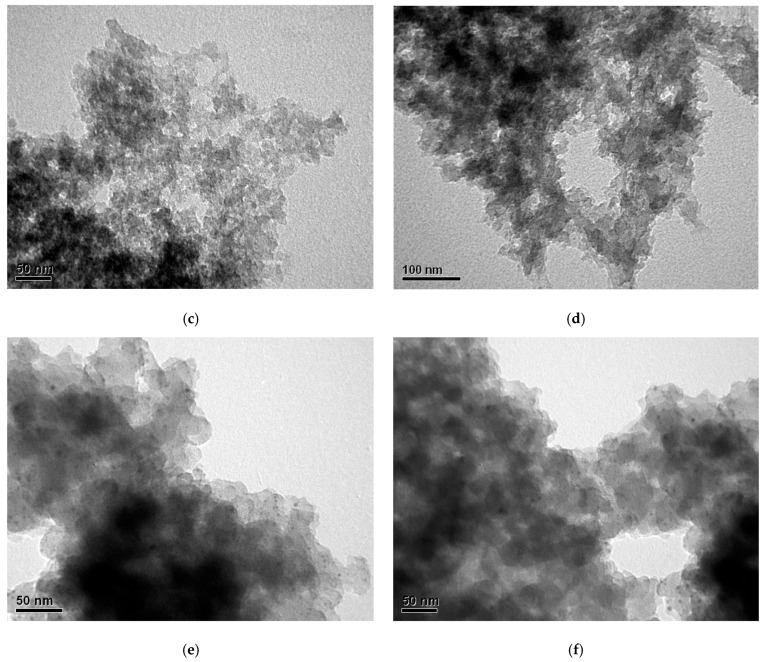
TEM micrographs for the used Pd-based catalysts. (**a**) Pd(CH_3_CO_2_)_2_), (**b**) Pd-SALOPHEN, (**c**) PdAS(1)-MA, (**d**) PdAS(2)-MA, (**e**) PdAS(5)-MA and (**f**) PdAS(10)-MA.

We found that a large I-Ph conversion was obtained for both Pd(CH_3_CO_2_)_2_ and PdAS(10)-MA, with the maximum conversion being reached after 30 min. Indeed, the catalytic results summarized in Table 3 indicate the dependence of the I-Ph conversion on the PdAS(x) content. This behavior can be explained by considering the catalytic mechanism of the Pd-catalyzed C–C cross-coupling reaction. The accepted catalytic cycle for the Heck reaction proposed by Richard F. Heck is summarized in Scheme 2 [7]. The superior catalytic activity exhibited by Pd(CH_3_CO_2_)_2_ was attributed to the formation of atomic Pd particles during the catalytic transformation. Indeed, a study by de Vries supported the formation of such particles, and this was explained by the fact that in the coupling of I-Ph with electron withdrawing olefins, alkene insertion is the rate determining step. When I-Ph is no longer present in the reaction media, Pd colloid formation takes place spontaneously [66]. This behavior was confirmed in our experiments by the detection of metallic Pd nano- and micro-particles after to the Heck reaction, as shown in Figure 8a.

However, the catalytic mechanism for the Pd-SALOPHEN catalysts could involve the formation of intermediates Pd(II)-Pd(0) [67,68], or different reaction states based on the participation of Pd(II)-Pd(IV) intermediates [69,70]. Recently, Venkateswarlu et al. proposed a modified mechanism for the Heck reaction, which involves the use of a Pd(II)-porphyrin tetradentate N_4_ complex [71], as shown in Scheme 2. This mechanism suggests the formation of Pd(0)-(L_2_) (i) from Pd(II)-(L_4_), either by dissociation followed by reduction or by reduction followed by dissociation. The coordinately unsaturated species, i, then undergoes oxidative addition with I-Ph to give intermediate ii, which undergoes insertion to cinnamate iii followed by β-hydrogen elimination to form iv. The intermediary iv participates in reductive elimination to regenerate the active component of Pd(0)-(L_2_) (i). Accordingly, since Pd(0)-(L_2_) is considered the catalytically active form of i in the Heck reaction, mercury poisoning tests [4] were carried out to evaluate the catalytic mechanism of the Pd-SALOPHEN homogeneous catalysts, as shown in of Figure 7b. The efficient catalytic inhibition observed following the addition of a large excess of Hg to the reaction demonstrates that the catalytic cycle of the homogenous Pd-SALOPHEN follows the Pd(II)-(L4)/Pd(0)-(L_2_) pathway. To confirm the mechanism proposed by Venkateswarlu et al., transmission electron microscopy (TEM) characterization of the catalytic medium was evaluated to determine the nature of the Pd-SALOPHEN catalysts post-reaction, as shown in Figure 8b. As no metallic Pd nanoparticles were detected after the I-Ph/MA coupling reaction, we propose that following the complete consumption of I-Ph, the Pd-SALOPHEN catalyst could be recovered to give the Pd(II)-(L_4_) form through the Pd(0)-(L_2_) ⇌ Pd(II)-(L_4_) equilibrium.

In the case of PdAS(1)-MA and PdAS(2)-MA, lower activities were observed for these catalysts compared with Pd-SALOPHEN due to the steric constraints provided by the resin influencing the geometry of the Pd-SALOPHEN complex, and consequently modifying the catalyst’s electronic and redox properties [67]. TEM characterization was then carried out on the used PdAS(1)-MA and PdAS(2)-MA catalysts, as shown in Figure 8c,d. Neither catalyst exhibited Pd NP formation following the reaction cycle, was it expected that the immobilized Pd-SALOPHEN complex on the MA-based resins followed the same catalytic cycle as the homogeneous catalyst at PdAS contents < 0.40 mmolg_solid_^−1^.

Interestingly, the PdAS(5)-MA catalyst exhibited a similar catalytic performance to the homogeneous Pd-SALOPHEN system. In this context, it is tempting to speculate that the PdAS(5)-MA catalyst, at this Pd-complex content, follows a similar reaction pathway during the Heck reaction as its Pd-SALOPHEN counterpart. Recently, Bi et al. reported the use of immobilized Pd-SALOPHEN on highly porous hyper-crosslinked polymers as catalysts for the Suzuki–Miyaura coupling reaction [31], and they found that the fresh catalysts suffered from Pd-metallization after continuous use. TEM analysis was therefore carried out for the used PdAS(5)-MA catalyst (Figure 8e), where the formation of small Pd-NPs with a narrow particle size distribution was observed. Furthermore, the PdAS(10)-MA catalyst exhibited an enhanced catalytic performance compared to Pd-SALOPHEN and the other PdAS(x)-MA heterogenous catalysts where x = 1, 2, or 5. To confirm the formation of Pd NPs on the catalyst surface, post reaction TEM was therefore carried out as shown in Figure 8f, and as expected, the formation of Pd-NPs on the PdAS(10)-MA surface was confirmed after the first reaction cycle.

As expected, an increase in the PdAS content of the resins altered the physicochemical properties and the catalytic activity of the immobilized Pd-complexes. Indeed, dispersion of the PdAS centers had a systematic influence on the catalytic performance, suggesting that in the case of PdAS(x)-MA where x = 5 or 10, the catalytically active species are Pd NPs. We therefore propose that with an increased PdAS incorporation, a decreased spacer content in the Pd(II)-(L_4_) species could be expected. When the PdAS(x)-MA with x ≥ 5 catalysts are submitted to the reaction conditions, the immobilized Pd(II)-(L_4_) pre-catalyst forms the Pd(0)-(L_2_) centers on the catalyst’s surface, the vicinal Pd(0)-(L_2_) species could coalescence by Ostwald ripening [5,40] to produce Pd-NPs which provoke a change in the catalytic performance in comparison with homogenous Pd-SALOPHEN, PdAS(2)-MA and PdAS(1)-MA resins catalysts. 

#### 3.8.2. Reusability

The catalyst reusability was then examined for PdAS(10)-MA over consecutive reaction cycles and stability testing was carried out in the post-reaction supernatant by AAS. The reusability test consisted of carrying out 5 consecutive cycles under the same reaction conditions, recovering the catalyst by filtration after each batch reaction, and evaluating the catalyst performance, morphology, and textural properties. 

**Figure 9:**
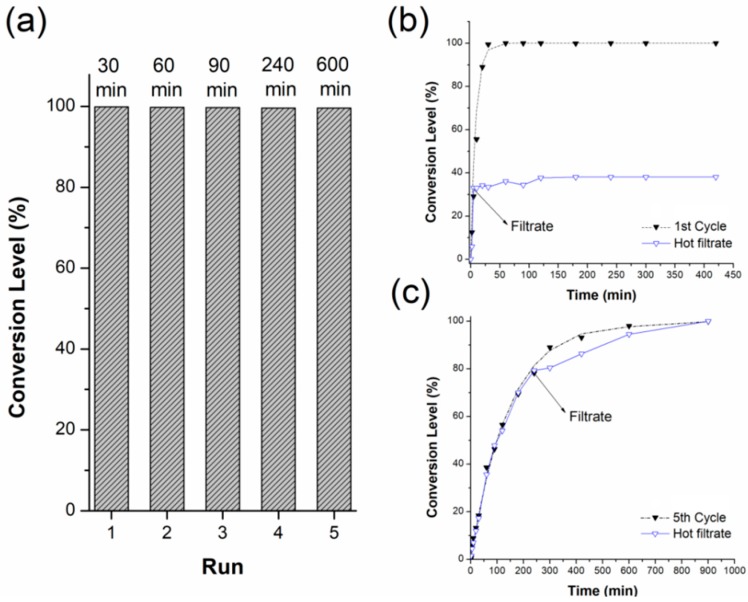
Recycle and hot filtration study for PdAS(10)-MA catalyst in the production of MCIN at 120 °C using DMF as solvent. (**a**) Recycles, (**b**) hot filtration test for the first cycle, and (**c**) hot filtration test for the fifth cycle.

Figure 9a shows the I-Ph conversion at the time of maximum conversion for each reaction cycle. A slight increase in time to reach the maximum conversion level was observed, indicating a small but progressive deactivation over five consecutive cycles. Moreover, hot filtration tests were carried out to evaluate the presence of soluble active species in the case of PdAS(10)-MA for the first and fifth cycles. The conversion profile shown in Figure 9b indicates that for the fresh PdAS(10)-MA catalyst, when the reaction was stopped after 5 min and the catalyst filtered, the I-Ph conversion was maintained at 37% for almost 5 h. In addition, for the fifth cycle, stopping the reaction and filtering after 240 min (Figure 9c) followed by maintaining the reaction conditions for 15 h allowed the contribution of the solubilized species to be detected.

**Figure 10:**
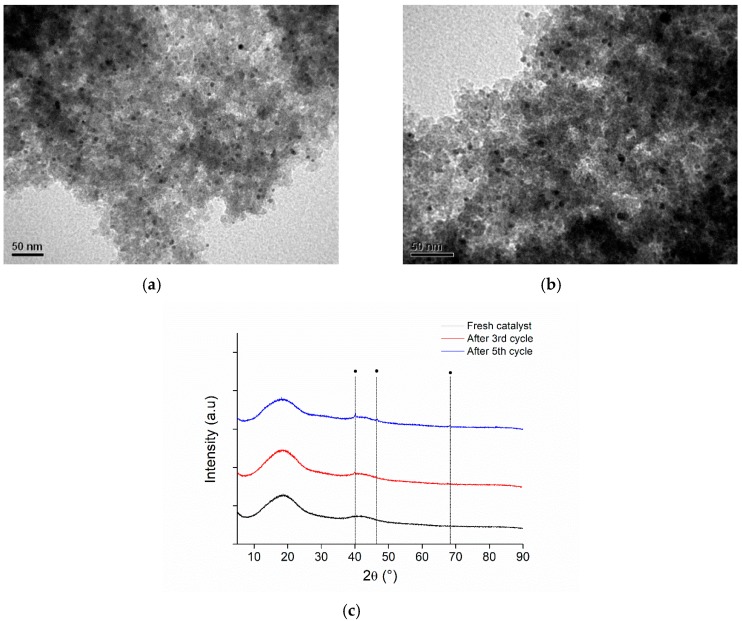
Physicochemical characterization of PdAS(10)-MA used catalyst. (**a**) TEM micrographs for PdAS(10)-MA catalyst after third reaction cycle, (**b**) TEM micrographs for PdAS(10)-MA catalyst after fifth reaction cycle, and (**c**) XRD diffraction pattern for the fresh catalyst, after third and fifth reaction cycle (● Metallic Pd (JCPDS 65-2867)).

Analyses by AAS, XRD, and TEM were then carried out for the used PdAS(10)-MA catalyst after the first, third, and fifth cycles, as shown in Figure 10. Leaching of the palladium species in the post-reaction liquid supernatant resulted in a maximum concentration of 4.6 wt.% Pd with respect to the total amount of Pd in the PdAS(10)-MA catalyst. However, XRD measurements for PdAS(10)-MA after the third and fifth cycles show the presence of typical diffraction peaks at 40.0, 46.4, and 68.2, which correspond to the (111), (200), and (220) planes of the Pd face-centered cubic (fcc) crystalline structure (JCPDS 65-2867), respectively. This result is in line with the TEM observations, where the Pd NPs showed an increase in their size distribution after continuous use. According to these results, the increase in the time required to reach the maximum conversion level for the catalyst could be attributed to the increase in the Pd NPs size.

## 4. Conclusions

We herein presented novel microsphere resins bearing well-defined Pd-SALOPHEN species on the surface, which were synthesized by a simple procedure to obtain high surface area polymer pre-catalysts with specific Brunauer–Emmett–Teller surface areas ≥ 580 m^2^·g^−1^, and which operate by a typical Pd(II)/Pd(0) Heck catalytic cycle. Spectroscopic measurements (X-ray photoelectron spectroscopy, infrared spectroscopy, and diffuse reflectance spectroscopy ultraviolet–visible light measurements) confirmed the presence of the expected complexes following the polymerization process. These novel solid-based microsphere resin catalysts were both highly active and selective in the Heck coupling of iodobenzene with methyl acrylate (MA) to produce methyl cinnamate as the reaction product. Consequently, this study clearly demonstrated that the catalytic effect of the palladium N,N’-bis(3-allylsalicylidene)o-phenylenediamine complex (PdAS) loading in the resin formulation had a systematic influence on the formation of Pd nanoparticles on the catalyst surfaces. More specifically, a nominal loading of ≥2.0 wt.% PdAS as a metal chelate monomer in the catalyst resulted in the irreversible formation of Pd nanoparticles on the catalyst surfaces. Indeed, we found that PdAS(10)-MA (10 wt.% PdAS) exhibited a superior catalytic performance, and due to its heterogeneous nature, it could be easily separated from the reaction mixture and reused after washing without any significant loss in activity over at least five consecutive reaction cycles. We therefore expect that the described immobilization of a homogeneous catalyst will lead to further advances in such materials for application in additional catalytic processes.

## Figures and Tables

**Table 1:** Nominal composition of divinylbenzene (DVB), methyl acrylate (MA), 2,2′-azobis(2-methylpropionitrile) (AIBN) and N,N’-bis(3-allyl-salicylidene)o-phenylenediamine palladium(II) (PdAS), scanning electron microscopy (SEM) grain diameter, surface area and pore size for PdAS(x)-MA polymers.

Label	wt.%				Grain Diameter ^1^ µm	S_BET_ m^2^g^−1^	Pore Size nm
	DVB	MA	AIBN	PdAS			
PdAS(1)-MA	60	38	1	1	184 ± 20	586	17.0
PdAS(2)-MA	60	37	1	2	196 ± 28	665	19.7
PdAS(5)-MA	60	34	1	5	227 ± 32	671	17.3
PdAS(10)-MA	60	29	1	10	272 ± 41	754	22.3

^1^ Based on the average pore diameter from Gaussian diameter distribution.

**Table 2:** Pd wt.% by inductively coupled plasma optical emission spectrometry (ICP-OES), Pd experimental/Pd nominal ratio, immobilized PdAS and X-ray photoelectron spectroscopy (XPS) core level of Pd 3d_5/2_ and N1s for PdAS(x)-MA polymers.

Label	Pd wt.%	Pd exp/Pd nom	Immobilized PdAS mmol g_cat_^−1^	Binding Energy eV	
				Pd 3d_5/2_	N 1s
PdAS	-	-	-	338.4	400.0
PdAS(1)-MA	0.36	1.3	0.034	338.0	400.4
PdAS(2)-MA	0.51	0.8	0.050	338.5	400.0
PdAS(5)-MA	0.77	0.6	0.074	338.5	400.0
PdAS(10)-MA	1.38	0.5	0.132	338.3	400.4

**Table 3:** I-Ph conversion, time to reach a conversion level of 5%, initial reaction rate (ro) and turnover frequency (TOF) for Pd-based catalysts.

Catalyst	Conversion %	Selectivity MCIN ^1^ %	t_5% conversion_^2^ min	r_o_ ×10^4^ mol·L^−1^min^−1^	TOF h^−1^
Pd(CH_3_CO_2_)_2_	100	100	0.2	1280	29554
PdAS	100	100	6.4	40	929
PdAS(1)-MA	58	100	43.7	2	137
PdAS(2)-MA	99	100	8.3	9	720
PdAS(5)-MA	99	100	2.2	67	2782
PdAS(10)-MA	100	100	1.0	265	6122

^1^ evaluated at the maximum of conversion level; ^2^ calculated according exponential fitting of [I-Ph] decrease included in the Figure S2 in supplementary material.

## Supplementary Materials

The following are available online at https://www.mdpi.com/1996-1944/12/16/2612/s1, Ligands and Pd-complexes synthesis, UV–vis of PdAS complex characterization, Scheme S1: Semi-Batch reactor used for the catalytic measurements, Figure S1: Microsphere diameter distribution of PdAS(x)-MA catalysts. (a) PdAS(1)-MA, (b) PdAS(2)-MA, (c) PdAS(5)-MA and (d) PdAS(10)-MA., Figure S2: Exponential fitting decrease curves of concentration of I-Ph on time for Pd-based catalysts. (a) Pd(CH_3_CO_2_)_2_, (b) Pd-SALOPHEN, (c) PdAS(1)-MA, (d) PdAS(2)-MA, (e) PdAS(5)-MA and (f) PdAS(10)-MA.
