# Supplementary material for: Mesoporous Palladium N,N’-Bis(3-Allylsalicylidene)o-Phenylenediamine-Methyl Acrylate Resins as Heterogeneous Catalysts for the Heck Coupling Reaction
Source: Materials (Basel). 2019 Aug 16;12(16):2612. doi: 10.3390/ma12162612 (PMC6721152; doi:10.3390/ma12162612)
Supplement: Supplementary file 1 [file materials-12-02612-s001.pdf]

materials

Supplementary

Mesoporous Palladium N,N′-Bis(3-Allylsalicylidene)o-Phenylenediamine -Methyl Acrylate Resins as Heterogeneous Catalysts for the Heck Coupling Reaction

Claudio Mella [1], Cecilia C. Torres [2], Gina Pecchi [1,3] and Cristian H. Campos [1,*]

[1] Depto. Físico-Química, Facultad de Ciencias Químicas, Universidad de Concepción, Edmundo Larenas 129, Concepción 4070371, Chile
[2] Depto. de Ciencias Químicas, Facultad de Ciencias Exactas, Universidad Andrés Bello, Sede Concepción, Autopista Concepción-Talcahuano 7100, Talcahuano 4300866, Chile
[3] Millenium Nuclei on Catalytic Processes towards Sustainable Chemistry (CSC), Santiago 8340518, Chile
* Correspondence: ccampos@udec.cl; Tel.: +56-41-220-3353

Received: 31 July 2019; Accepted: 14 August 2019; Published: 16 August 2019

1. Ligands and Pd-Complexes Synthesis

1.1. Synthesis of SALOPHEN Ligand

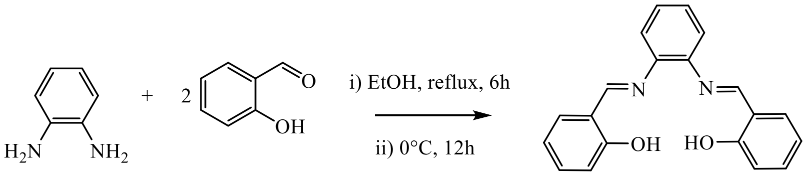

The ligand N,N′-bis(salicylidene)o-phenylenediamine (SALOPHEN) was synthesized according to a reported procedure [1]. A 250-mL round-bottomed flask was charged with salicaldehyde (40.0 mmol) in EtOH, and a solution with o-phenylenediamine (20.0 mmol) in 50 mL of EtOH was added to it dropwise. The reaction mixture was then refluxed for 2 h with magnetic stirring. After the reaction, the mixture was cooled at 0 °C for 12 h to obtain a precipitate, which was recrystallized repeatedly from EtOH until the purified ligand AS was obtained as an orange well-defined solid with 78% yield. ^{1}H NMR (400 MHz, $CDCl_3$): δ 13.04 (s, 2H, –OH); 8.65 (s, 2H, –N=C–H); 7.38 (m, 6H, H-Ar); 7.36–7.24 (m, 4H, H-Ar); 7.06 (d, 2H, H-Ar); and 6.93 (t, 2H, H-Ar). ^{13}C NMR (75 MHz, $CDCl_3$): δ 164.90 (Ar-OH); 150.60 (Ar-C=N); 142.05; 135.98; 134.71; 126.95; 120.08; 119.60; 116.91; 114.95 (Aromatic carbons).

1.2. Synthesis of AS ligand

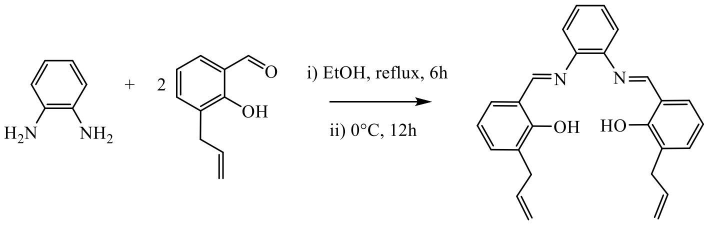

The ligand N,N′-bis(3-allylsalicylidene)o-phenylenediamine (AS) was synthesized follow the same procedure as a SALOPHEN ligand using 3-allylsalicaldehyde instead of 3-salicaldehyde. After the reaction, the mixture was cooled at 0 °C for 12 h to obtain a precipitate, which was recrystallized repeatedly from EtOH until the purified ligand AS was obtained as an orange well-defined solid with 81% yield. ^{1}H NMR (400 MHz, DMSO d_6): δ 13.51 (s, 2H, –OH); 8.94 (s, 2H, –N=C–H); 7.50 (m, 4H, H-

Ar); 7.43 (m, 2H, H-Ar); 7.29 (d, 2H, H-Ar); 6.93 (2H, H-Ar); 6.02 (m, 2H, –HC=C–); 5.08 (dd, 2H, HC=C–); 5.0 (dd, 2H, -HC=C–), and 3.49 (d, 4H, Ar-H_2C–C=C). ^{13}C NMR (75 MHz, DMSO d_6): δ 165.90 (Ar-O); 152.49 (Ar-C=N); 143.64; 135.30; 133.60; 132.72; 127.65; 120.01; 115.86; 115.60; y 115.48 (Aromatic carbons); 137.30 (C=CH–), (H_2C=C–), 35.90 (–CH_2–) corresponding to allyl group.

1.3. Synthesis of Pd-SALOPHEN Complex

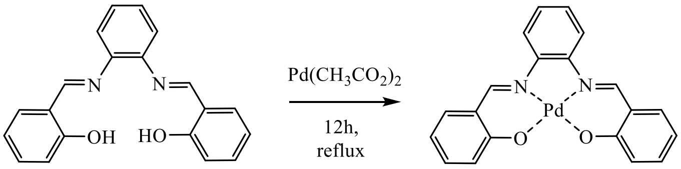

The SALOPHEN ligand (15 mmol) was added to 150 mL of DCM and stirred at room temperature, followed by adding Pd(CH_3COO)$_2$ (15.5 mmol) to the mixture and refluxing for 12 h [2,3]. A brown precipitate was separated by filtration and washed using MeOH and DCM (4 × 5 mL). 1H NMR (400 MHz, DMSO d_6): δ 9.19 (s, 2H, –N=C–H); 8.34 (m, 2H, H-Ar); 7.74 (d, 2, H-Ar); 7.45 (m, 4, H-Ar); 7.03 (d, 2H, H-Ar); 6.72 (t,2H, H-Ar). ^{13}C NMR (75 MHz, DMSO d_6): δ 165.80 (Ar-O); 153.61 (Ar-C=N); 142.85; 136.35; 135.80; 127.90; 120.73; 120.40; 117.15; 115.51 (Aromatic carbons).

1.4. Synthesis of Pd-AS Complex

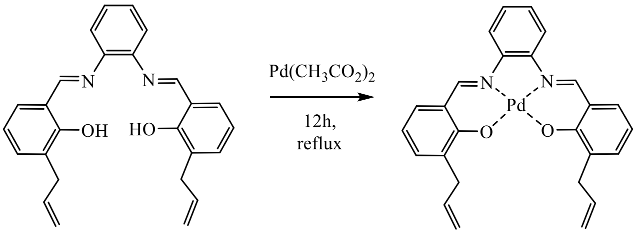

The homogeneous catalysts PdAS was synthesized in the same way as Pd-SALOPHEN was prepared, producing a brown solid with 80% yield. 1H NMR (400 MHz, DMSO d_6): δ 8.41 (s, 2H, –N=C–H); 7.78 (dq, 2H, H-Ar); 7.30–7.25 (m, 4H, H-Ar); 6.62 (dd,2H, H-Ar); 6.15 (m, 2H, –HC=C–); 5.28 (dd, 2H, HC=C–); 5.25 (dd, 2H, –HC=C–); and 3.59 ppm (d, 4H, Ar-H_2C–C=C). ^{13}C NMR (75 MHz, DMSO d_6): δ 165.90 (Ar-O); 152.49 (Ar-C=N); 143.64; 135.30; 133.60; 132.72; 127.65; 120.01; 115.86; 115.60; y 115.48 (Aromatic carbons); 137.30 (C=CH–), (H_2C=C–), 35.90 (–CH_2–) corresponding to allyl group.

materials

2. UV-Vis of PdAS Complex Characterization

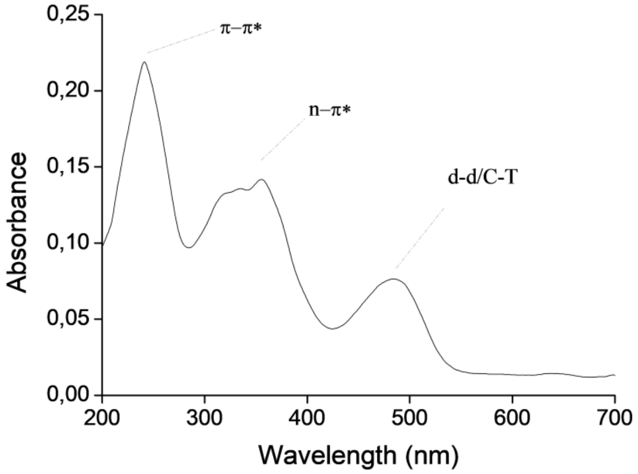

The UV–visible spectra in liquid phase showed the transition bands $\pi \rightarrow \pi$ * at 241 nm, n $\rightarrow \pi$ * between 336 and 355 nm and charge–transfer (C–T) transition at 481 nm, in agreement with results reported by Choudhary et al. [4].

3. Schemes

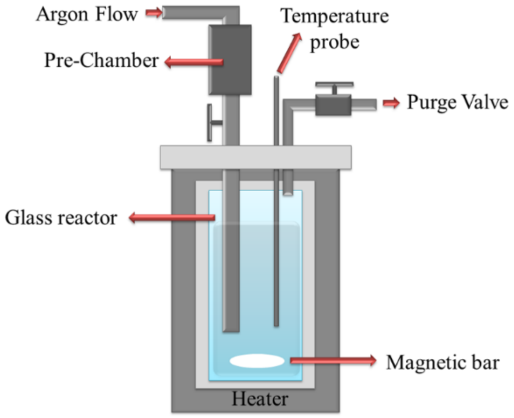

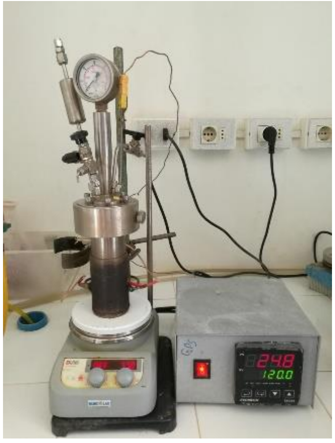

Scheme S1. Semi-Batch reactor used for the catalytic measurements.

materials

4. Complementary PdAS(x)-MA Catalysts Characterization

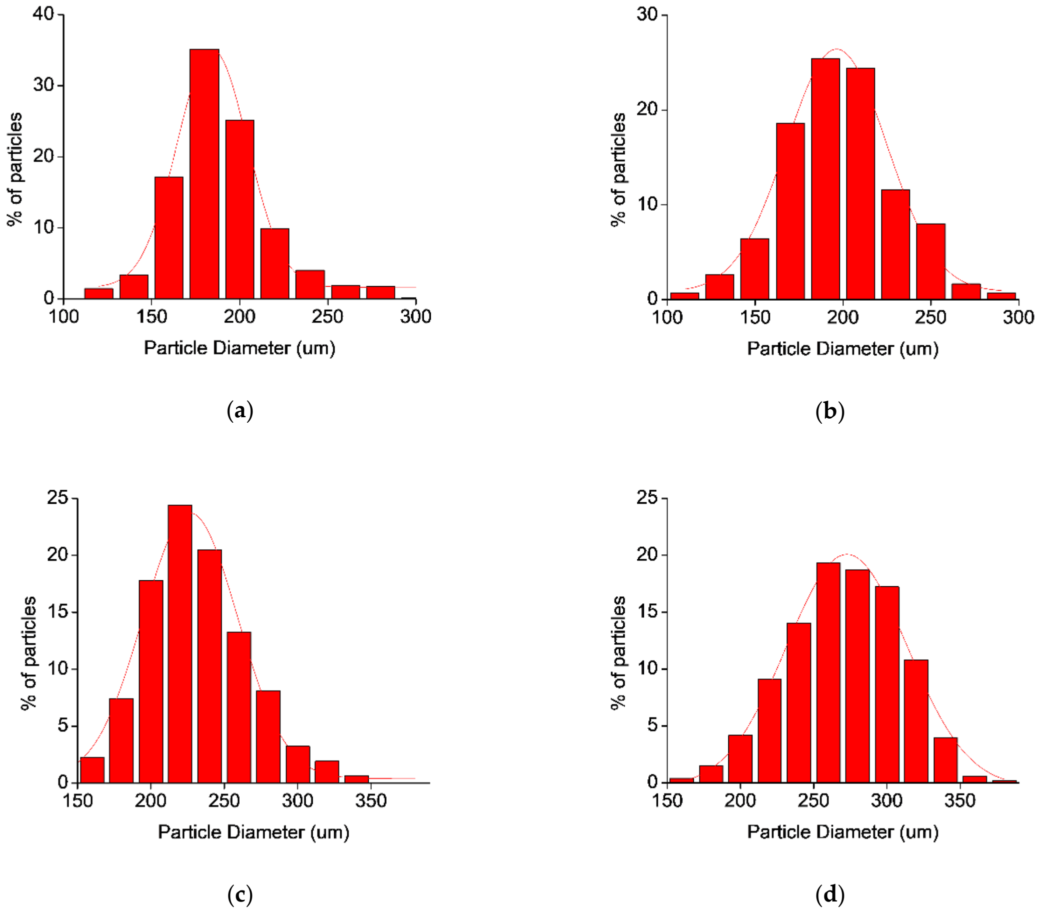

Figure S1. Microsphere diameter distribution of PdAS(x)-MA catalysts. (**a**) PdAS(1)-MA, (**b**) PdAS(2)-MA, (**c**) PdAS(5)-MA, and (**d**) PdAS(10)-MA.

(a)

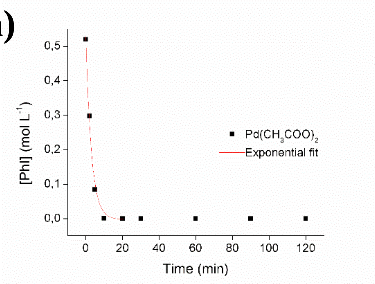

*y = y0 + A*exp(R0*x)*

Adj. R-Square 0.995

	Value	*Standard Error*
y0	*−0.00231*	*0.00365*
A	*0.5302*	*0.0114*
R0	*-0.32536*	*0.01686*

(b)

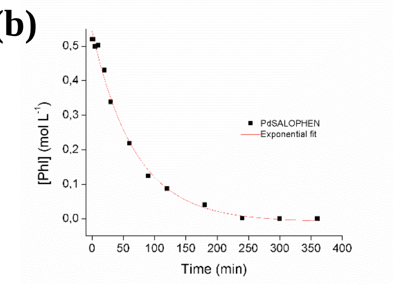

*y = y0 + A*exp(R0*x)*

Adj. R-Square 0.99384

	Value	*Standard Error*
y0	*−0.00858*	*0.01094*
A	*0.55206*	*0.01288*
R0	*−0.01454*	*0.00104*

(c)

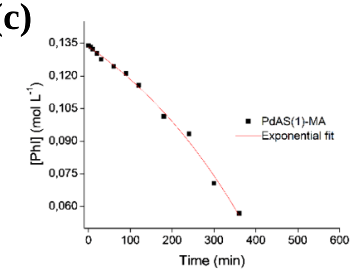

*y = y0 + A*exp(R0*x)*

Adj. R-Square 0.99416

	Value	*Standard Error*
y0	*0.18613*	*0.01582*
A	*−0.0527*	*0.01523*
R0	*0.00252*	*4.88351E-4*

(d)

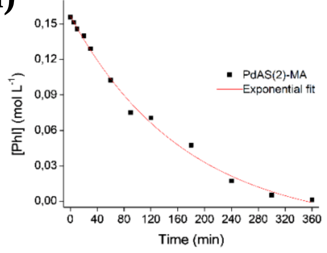

*y = y0 + A*exp(R0*x)*

Adj. R-Square 0.99393

	Value	*Standard Error*
y0	*−0.02314*	*0.00868*
A	*0.17985*	*0.00799*
R0	*−0.00582*	*6.24366E-4*

(e)

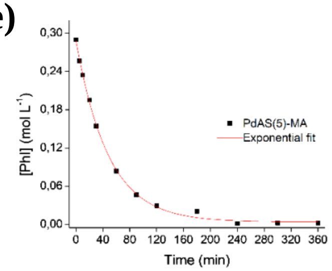

*y = y0 + A*exp(R0*x)*

Adj. R-Square 0.99844

	Value	*Standard Error*
y0	*0.00395*	*0.00226*
A	*0.28397*	*0.0034*
R0	*−0.02074*	*7.16161E-4*

(f)

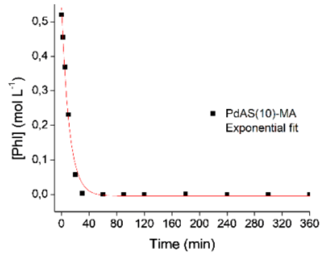

*y = y0 + A*exp(R0*x)*

Adj. R-Square 0.99197

	Value	*Standard Error*
y0	*−0.00475*	*0.00653*
A	*0.54475*	*0.01505*
R0	*−0.09003*	*0.00648*

Figure S2. Exponential fitting decrease curves of concentration of I-Ph on time for Pd-based catalysts. (**a**) $Pd(CH3CO_2)_2$, (**b**) Pd-SALOPHEN, (**c**) PdAS(1)-MA, (**d**) PdAS(2)-MA, (**e**) PdAS(5)-MA, and (**f**) PdAS(10)-MA.

References

1. He, F.F.; Wang, H.Q.; Wang, Y.Y.; Wang, X.F.; Zhang, H.S.; Li, H.L.; Tang, J.H. Magnetic Th (IV)-ion imprinted polymers with salophen schiff base for separation and recognition of Th (IV). *J. Radioanal. Nucl. Chem.* **2013**, *295*, 167–177.
2. Soh, C.; Kamilah, S.; Shamsuddin, M. Tetradentate N_2O_2 Chelated Palladium (II) Complexes: Synthesis, Characterization, and Catalytic Activity towards Mizoroki-Heck Reaction of Aryl Bromides. *J. Chem.* **2013**, *2013*, 632315.
3. Tobiasz, A.; Walas, S.; Trzewik, B.; Grzybek, P.; Zaitz, M.M.; Gawin, M.; Mrowiec, H. Cu (II)-imprinted styrene-divinylbenzene beads as a new sorbent for flow injection-flame atomic absorption determination of copper. *Microchem. J.* **2009**, *93*, 87–92.
4. Choudhary, A.; Kumari, S.; Ray, S. Tuning of Catalytic Property Controlled by the Molecular Dimension of Palladium-Schiff Base Complexes Encapsulated in Zeolite Y. *ACS Omega* **2017**, *2*, 6636–6645.

© 2019 by the authors. Submitted for possible open access publication under the terms and conditions of the Creative Commons Attribution (CC BY) license (http://creativecommons.org/licenses/by/4.0/).
